# USP15 promotes pulmonary vascular remodeling in pulmonary hypertension in a YAP1/TAZ-dependent manner

**DOI:** 10.1038/s12276-022-00920-y

**Published:** 2023-01-12

**Authors:** Zhuhua Wu, Li Zhu, Xinran Nie, Li Wei, Yong Qi

**Affiliations:** 1 Department of Pulmonary and Critical Care Medicine, Zhengzhou University People’s Hospital, Henan Provincial People’s Hospital, Zhengzhou, Henan China; 2 Department of Thoracic Surgery, Zhengzhou Key Laboratory for Surgical Treatment for End-Stage Lung Disease, Henan Provincial People’s Hospital, Zhengzhou University People’s Hospital, Zhengzhou, Henan China

**Keywords:** Ubiquitylation, Diseases

## Abstract

Pulmonary hypertension (PH) is a life-threatening cardiopulmonary disease characterized by pulmonary vascular remodeling. Excessive growth and migration of pulmonary artery smooth muscle cells (PASMCs) are believed to be major contributors to pulmonary vascular remodeling. Ubiquitin-specific protease 15 (USP15) is a vital deubiquitinase that has been shown to be critically involved in many pathologies. However, the effect of USP15 on PH has not yet been explored. In this study, the upregulation of USP15 was identified in the lungs of PH patients, mice with SU5416/hypoxia (SuHx)-induced PH and rats with monocrotaline (MCT)-induced PH. Moreover, adeno-associated virus-mediated functional loss of USP15 markedly alleviated PH exacerbation in SuHx-induced mice and MCT-induced rats. In addition, the abnormal upregulation and nuclear translocation of YAP1/TAZ was validated after PH modeling. Human pulmonary artery smooth muscle cells (hPASMCs) were exposed to hypoxia to mimic PH in vitro, and USP15 knockdown significantly inhibited cell proliferation, migration, and YAP1/TAZ signaling in hypoxic hPASMCs. Rescue assays further suggested that USP15 promoted hPASMC proliferation and migration in a YAP1/TAZ-dependent manner. Coimmunoprecipitation assays indicated that USP15 could interact with YAP1, while TAZ bound to USP15 after hypoxia treatment. We further determined that USP15 stabilized YAP1 by inhibiting the K48-linked ubiquitination of YAP1. In summary, our findings reveal the regulatory role of USP15 in PH progression and provide novel insights into the pathogenesis of PH.

## Heart disease: identifying a key enzyme in pulmonary hypertension

An enzyme that plays a key role in a serious heart condition provides insights into disease progression and possible treatment routes. Pulmonary hypertension occurs when the walls of the pulmonary arteries, which deliver blood from the heart to the lungs, thicken and stiffen due to excessive growth of smooth muscle cells (PASMCs), increasing pressure in the arteries. Yong Qi and Li Wei at Zhengzhou University People’s Hospital in Zhengzhou, China, and co-workers examined the role of the ubiquitin-specific protease 15 (USP15) enzyme. This enzyme was significantly increased in samples from the lungs of pulmonary hypertension patients and animal models. USP15 regulates a key signaling pathway, stabilizing one component of the pathway to actively boost the proliferation of PASMCs. Blocking USP15 expression in cell cultures inhibited PASMC growth and reduced disease progression.

## Introduction

Pulmonary hypertension (PH) is a progressive and devastating cardiopulmonary disease clinically defined by a mean pulmonary artery pressure of 25 mmHg or above at rest^1^. On the basis of its etiology or comorbidity, PH is categorized into five groups: pulmonary arterial hypertension (PAH), PH caused by left-side heart disease, PH caused by hypoxia/chronic lung disease, chronic thromboembolic PH, and PH with unclear mechanisms^2^. Currently, several therapies have been developed to improve PH, such as endothelin receptor antagonists, phosphodiesterase inhibitors, and prostacyclin analogs^3,4^. However, the therapeutic outcomes of PH are still not satisfactory. PH patients always have a poor prognosis. Thus, it is necessary to further investigate the pathogenesis of PH and find novel therapeutic targets for PH.

Reportedly, the remodeling of pulmonary arteries is a key feature of PH, which progressively results in the elevation of pulmonary artery resistance^5^. Notably, excessive cell proliferation and migration of pulmonary artery smooth muscle cells (PASMCs) are essential events during the process of pulmonary artery remodeling^6^. Pioneer studies suggest that intervention of excessive proliferation in PASMCs might be a promising strategy to prevent pulmonary artery remodeling in PH^7,8^, which makes PASMCs a key factor in PH pathogenesis.

Ubiquitin-specific protease 15 (USP15) belongs to the superfamily of deubiquitinases (DUBs), which regulate ubiquitin-mediated signaling by catalyzing the removal of ubiquitin from substrate proteins^9^. USP15 promotes tumor cell survival in several human malignancies^10,11^, and its functional deficiency significantly reversed mitophagy defects in Parkinson’s disease (PD) patients^12^. In addition, USP15 exhibits a regulatory role in osteoarthritis by regulating TGF-β/SMAD2 signaling^13^. However, the effect of USP15 on PH has not yet been explored. Microarray data from the Gene Expression Omnibus (GEO) database (GSE113439, GSE15197, and GSE53408) identified the upregulation of USP15 in lung tissues of PAH patients, which suggests the potential contribution of USP15 in PH. Strikingly, USP15 has been validated to facilitate cell proliferation and migration of hypertrophic scar-derived fibroblasts in vivo^14^. We thus hypothesize that USP15 might participate in the process of PASMC proliferation and migration in PH.

Yes-associated protein (YAP)-1 and tafazzin (TAZ) are two key transcriptional regulators of the Hippo pathway^15,16^. These molecules have shown profound effects on tumor development and cardiac biology^16–18^. Additionally, the Hippo-YAP1/TAZ axis is capable of controlling organ size by regulating cell proliferation, differentiation, and apoptosis^19^. More surprisingly, YAP1/TAZ signaling was evidently activated in PASMCs from experimental PH^20^, and YAP1 interference prominently suppressed the proliferation of vascular smooth muscle cells in vitro^21,22^, suggesting the contribution of YAP1 to PASMC proliferation during PH progression. The HitPredict database (http://www.hitpredict.org/) further predicts the interaction between USP15 and YAP1. Therefore, our study aims to investigate whether USP15 promotes pulmonary vascular remodeling in PH via YAP1/TAZ signaling.

## Materials and methods

### GEO database

Microarray data from the GEO database (GSE113439, GSE15197, and GSE53408) were used to identify aberrantly expressed USP15 in the lungs of PAH patients. Gene Ontology (GO) and Kyoto Encyclopedia of Genes and Genomes (KEGG) enrichment analyses were further conducted for functional analysis.

### Human specimen collection

Human lung tissues from patients with or without PH were collected from Henan Provincial People’s Hospital. This study was performed according to the principles in the Declaration of Helsinki and approved by the Medical Ethics Committee of Henan Provincial People’s Hospital (Ethical Review 2021(60)). Written informed consent from all participants was provided for the use of their lung tissues. For the criteria of PH diagnosis in this study, the pulmonary artery pressure of patients was detected by experienced sonographers via echocardiography. The systolic pulmonary artery pressure (sPAP) was calculated by the Bernoulli equation (maximum tricuspid regurgitant velocity (TRV) plus estimated right atrial pressure). Individuals with sPAP > 30 mmHg were diagnosed with PH. Human lung tissues were obtained from patients with end-stage lung diseases undergoing lung transplantation. The patients were divided into a control group (*n* = 3) and a PH group (*n* = 6) according to the presence or absence of PH.

### Chemicals

The vascular endothelial growth factor receptor (VEGF) inhibitor SU5416 (204005-46-9) and cycloheximide (CHX, C112766) were purchased from Aladdin reagent. MG132 (HY-13259) was purchased from MCE. Monocrotaline (MCT, C835791) was purchased from Macklin reagent.

### Animal study

The animal experiments involved in this study were conducted in accordance with the guidelines for the care and use of experimental animals. All procedures involving animals were approved by the Animal Ethics Committee of Henan Provincial Institute for Food and Drug Control (No. YXKC2020005-1). All experimental animals were adaptively housed for a week with a temperature of 22 ± 1 °C, humidity of 45–55%, 12-h day/night cycle, and free access to water and food.

Experiment 1: C57BL/6 J mice (8 weeks old) were randomly divided into two groups (*n* = 8 per group): the sham and SuHx-PH groups. The SuHx-PH mice received a weekly subcutaneous injection of the VEGFR inhibitor SU5416 (20 mg/kg, Aladdin reagent) and were exposed to hypoxia (10% O_2_, 90% N_2_) for 5 weeks^23^. The sham mice received weekly injections with the same volume of the solvent and were exposed to normoxia for 5 weeks.

Experiment 2: For investigation of the preventative effect of USP15 knockdown on the SuHx-induced PH model, C57BL/6 J mice (8 weeks old) were randomly divided into 5 groups (*n* = 8 per group): sham, SuHx-PH, SuHx-PH + NCsh-1-p, SuHx-PH + mUSP15-sh1-p and SuHx-PH + mUSP15-sh2-p. Fourteen days prior to modeling, the mice in the latter 3 groups were intravenously injected with the corresponding adeno-associated virus (AAV, 1 × 10^12^ vector genomes/mL) carrying shRNAs against mUSP15 or negative control (NC). Five weeks after SuHx induction, right ventricular systolic pressure (RVSP) was measured with standard protocols^24^. The ratio of the weight of the right ventricle to the weight of the left ventricle plus septum (RV/LV + S) was subsequently determined to identify right ventricular hypertrophy.

Experiment 3: For analysis of the reversal effect of USP15 knockdown on the SuHx-induced PH model, C57BL/6 J mice (8 weeks old) were randomly divided into 4 groups (*n* = 8 per group): sham, SuHx-PH, SuHx-PH + NCsh-1-r and SuHx-PH + mUSP15-sh2-r. After 5 weeks of SuHx induction, mice in the AAV-treated group were intravenously injected with the corresponding AAVs (1 × 10^12^ vector genomes/mL) for 4 weeks. RVSP and RV/LV + S ratio were subsequently determined as previously described.

Experiment 4: For investigation of the preventative/reversal effect of USP15 knockdown on the MCT-induced PH model, Sprague‒Dawley (SD) rats (male, 10-14 weeks old) were randomly divided into 6 groups: sham, MCT, MCT + NCsh-1-p, MCT + RUSP15-sh-p, MCT + NCsh-1-r, and MCT + RUSP15-sh-r. The rats in the MCT-treated group were subcutaneously injected with 60 mg/kg MCT once (Macklin, Shanghai) and maintained for 4 weeks. For the preventative model, rats were intravenously injected with AAVs carrying shRNAs against RUSP15 and NC 14 days prior to MCT induction. For the reversal model, rats received an intravenous injection of the corresponding AAVs 4 weeks after MCT injection and were maintained for another 4 weeks. RVSP and the RV/LV + S ratio were subsequently determined as previously described.

### Recombinant virus generation

AAV vectors carrying mUSP15/RUSP15 shRNA and NC sequences were generated using the established packing system^25^. The indicated amounts of AAVs were used to infect experimental animals by tail intravenous injection. Adenoviruses (Ad) expressing hUSP15-OV, hUSP15-sh, hYAP1-sh, and hTAZ-sh were packaged and used to infect the indicated cells. The recombinant virus particles carrying their NC sequence were produced as the control. shRNA sequence information is provided in Table 1, and all plasmids were validated by DNA sequencing.

**Table 1:** shRNA sequence information.

Name	Target sequence (5′–3′)
mUSP15-sh1	GCTGACACAATAGATACGATT
mUSP15-sh2	TGAGAGGTGAAATAGCTAAA
hUSP15-sh	TGAGAGGTGAAATAGCTAAA
hYAP1-sh	GCCACCAAGCTAGATAAAGAA
hTAZ-sh	CGGACTTCATTCAAGAGGAAT

### Cell culture

Human pulmonary artery smooth muscle cells (hPASMCs) were purchased from iCell Bioscience, Inc., Shanghai. hPASMCs were cultured in specific medium (PriMed-iCell-004-LS, iCell Bioscience, China) at 37 °C in a humidified incubator with 5% CO_2_. hPASMCs at passages 2-7 were used for further study. The human embryonic kidney cell line HEK293T was obtained from Shanghai Zhongqiaoxinzhou Biotech and cultured in DMEM (G4510, Servicebio, China) with 10% FBS (11011-861, Tianhang Biotechnology, Zhejiang) at 37 °C in a humidified incubator with 5% CO_2._

### H&E staining

Lung tissues were fixed, paraffin-embedded, and sliced into 5-μm sections. Sections were further stained with hematoxylin (H8070, Solarbio) and eosin (A600190, Sangon Biotech) using established protocols^7^. Finally, the sections were sealed and observed under a microscope (BX53, Olympus, Japan). The ratio of vascular medial thickness to cross-sectional area (Medial/Cross-Sectional Area) was analyzed to show the degree of medial wall thickness^26^.

### Immunofluorescence staining

For the immunofluorescence assay, lung sections were prepared according to the established protocol^3,27^. Slides from lung tissues were coincubated with primary antibodies against USP15/α-SMA, USP15/YAP1, USP15/TAZ, and Ki-67/α-SMA at 4 °C overnight to determine the colocalization of target proteins. Incubation with FITC-labeled goat anti-rabbit IgG (A0562, Beyotime, China) and Cy3-conjugated goat anti-mouse IgG (A0521, Beyotime, China) was subsequently conducted in the dark at room temperature for 90 min, followed by DAPI staining and image capture. The degree of pulmonary arteriolar muscularization in lung sections stained with α-SMA was analyzed by calculating the proportion of muscularized pulmonary arterioles to the total as previously described^24^. Immunofluorescence staining was performed to determine the expression of α-SMA and desmin in hPASMCs. After fixing and permeabilization, the cells were incubated with primary antibodies against α-SMA and desmin at 4 °C overnight. The cells were then probed with the corresponding secondary antibodies and restained with DAPI (D106471-5 mg, Aladdin reagent, China). Subsequently, representative images were captured under a fluorescence microscope (BX53, Olympus, Japan). The antibody information is listed in Table 2.

**Table 2:** Primary antibodies used in the study.

Antibody	Application:	Cat. No.	Manufacturer
α-SMA	IF	19245	CST
USP15	IF, WB, IP	sc515688	Santa Cruz
YAP1	IF	AF6328	Affinity
TAZ	IF	DF4653	Affinity
Ki-67	IF	A2094	Abclonal
Desmin	IF	AF5334	Affinity
YAP1	WB, IP	13584-1-AP	Proteintech
TAZ	WB, IP	23306-1-AP	Proteintech
PCNA	WB	A12427	Abclonal
His	WB, IP	AE003	Abclonal
Myc	WB, IP	AE070	Abclonal
K48-Ub	WB	A3606	Abclonal
Histone H3	WB	17168-1-AP	Proteintech
Actin	WB	60008-1-Ig	Proteintech

### Cell Counting Kit-8 assay

Cell proliferation was assessed by a Cell Counting Kit-8 (CCK-8, KGA317, KeyGen Biotech, China) assay. In brief, pretreated hPASMCs were exposed to hypoxia (2% O_2_, 93% N_2_ and 5% CO_2_) or normoxia for the indicated times. CCK-8 (10 μL/well) was subsequently added, and the cells were further cultured at 37 °C for 2 h in a humidified incubator. Absorbance at 450 nm was detected by a microplate reader (800TS, Biotek, USA).

### Wound healing assay

A scratch assay was conducted to determine the migratory ability of hPASMCs in each group. Briefly, pretreated cells were incubated with serum-free medium with mitomycin C (1 μg/ml, M0503, SIGMA) for 1 h. Subsequently, a scratch was made in the cells using a pipette tip. Afterward, the cells were exposed to hypoxia (2% O_2_, 93% N_2_, and 5% CO_2_) or normoxia for 24 h. Representative images were captured at time points of 0 h and 24 h to measure cell migration.

#### Real-time PCR

Total RNA from lung tissues and hPASMCs was extracted by TRIpure reagent (RP1001, BioTeke, China). cDNAs were synthesized using reverse transcriptase (D7160L, Beyotime, China). Real-time PCR analysis was further performed on an Exicycler®96 (Bioneer, Korea) to detect the relative expression of target genes using SYBR Green Mix (SY1020, Solarbio, China), cDNAs, and corresponding primers. Gene expression was normalized to β-actin and analyzed by the 2^−ΔΔCt^ method. The primers used in this study were synthesized in GenScript, and the primer sequences are provided in Table 3.

**Table 3:** Real-time PCR primer information.

Gene	Sequence (5′-3′)	Product length (bp)
HOMO USP15 F	GCTTTGACAGTTGGGACA	215
HOMO USP15 R	CACCTTTCGTGCTATTGG	
MUS USP15 F	GATGGAAACCGACGAGC	296
MUS USP15 R	CCCAGTCCAAAGCGAGA	
MUS TAZ F	CTCCCTAACAGCCCACCCT	132
MUS TAZ R	TTTCCGCATCTCCACAGC	
MUS YAP1 F	GGCAGGCAATACGGAATA	224
MUS YAP1 R	GCCGCTGTCTGTGCTCT	
HOMO YAP1 F	GCAATGCGGAATATCAATC	186
HOMO YAP1 R	GGTGCCACTGTTAAGGAAAG	
HOMO TAZ F	TTCCTAACAGTCCGCCCTAC	131
HOMO TAZ R	TTTCCGCATCTCCACAGC	

### Western blotting

Total protein from lung tissues and hPASMCs was isolated using RIPA buffer (P0013B, Beyotime, China) with 1% PMSF (ST506, Beyotime, China). Nuclear protein was extracted using a nuclear protein extraction kit (P0028, Beyotime, China). Protein concentration was measured by a BCA kit purchased from Beyotime Institute of Biotechnology (P0009, Haimen, China). Protein samples were loaded on SDS-PAGE gels and transferred onto polyvinylidene difluoride (PVDF) membranes. After blocking, the membranes were incubated with primary antibodies against USP15 (1:200, sc515688, Santa Cruz), YAP1 (1:1000, 13584-1-AP, Proteintech), TAZ (1:1000, 23306-1-AP, Proteintech), and PCNA (1:1000, A12427, Abclonal) at 4 °C overnight. Histone H3 (1:500, 17168-1-AP, Proteintech) and actin (1:2000, 60008-1-Ig, Proteintech) were used as internal controls. After 4 washes (5 min/wash), the membranes were probed with secondary antibodies (1:10000, SA00001-2, SA00001-1, Proteintech) at 37 °C for 40 min. After 4 thorough washes, the protein bands were developed by ECL solution (E003, 7 Sea Biotech, China) and quantified using Gel-Pro-Analyzer software.

### Coimmunoprecipitation assay

A coimmunoprecipitation (Co-IP) assay was conducted using a commercial Co-IP kit (#26149, Pierce). In brief, cell lysate for Co-IP was isolated using a specific extraction reagent (P0013, Beyotime, China). Subsequently, purified cell lysates were incubated with antibody-conjugated resin at 4 °C overnight. After elution, protein samples were subjected to western blot analysis using the indicated antibody.

### Statistical analysis

The results are expressed as the mean ± SD. One-way ANOVA followed by Tukey’s post hoc test was used in most cases. An unpaired Student’s *t* test was also performed when appropriate. For all analyses, *P* < 0.05 was deemed statistically significant. GraphPad Prism software was used for data analyses.

## Results

### USP15 was overexpressed in lung tissues of patients with PH

**Fig. 1:**
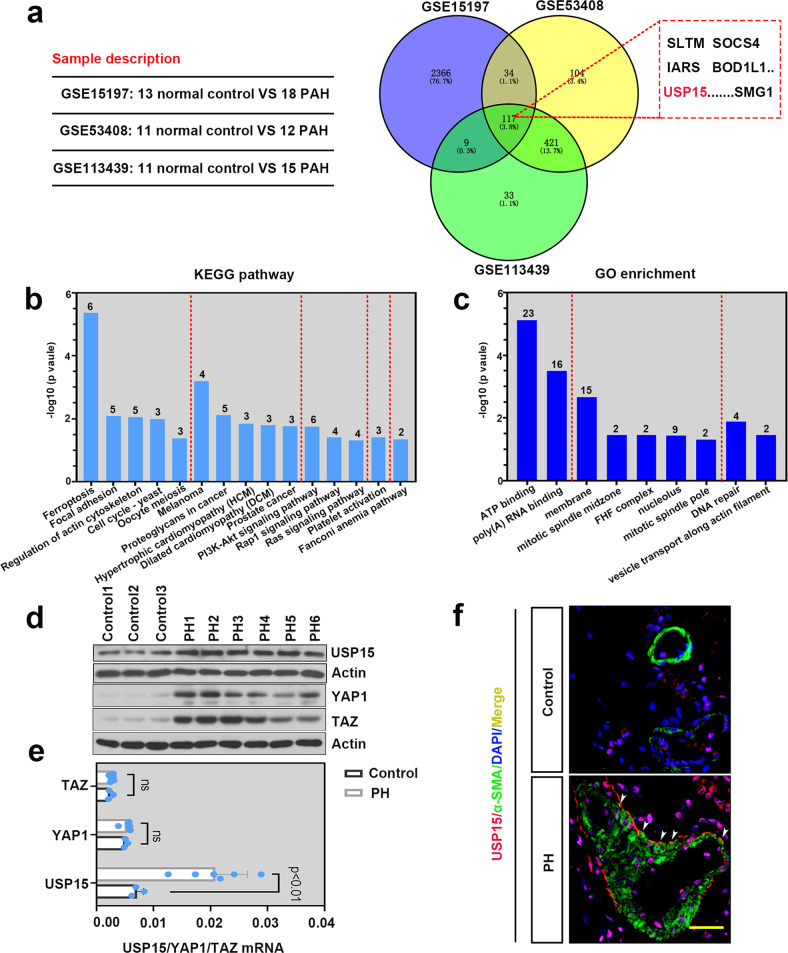
USP15 was overexpressed in the lung tissue of PH patients. Three mRNA expression datasets (GSE113439, GSE15197, and GSE53408) were selected to identify DEGs between PAH lung specimens and normal lung specimens. **a** Overlap of these DEGs are represented by Venn diagrams. **b**–**c** DEGs were subjected to GO and KEGG enrichment analyses for functional analysis. The expression levels of USP15, YAP1, and TAZ in PH lung specimens (*n* = 6) and non-PH lung tissue (*n* = 3) were further validated by western blotting (**d**) and real-time PCR (**e**). **f** An immunofluorescence assay was conducted to investigate the expression of USP15 (red) and α-SMA (green) in lung tissue from PH patients (*n* = 1) and controls (*n* = 1). Scale bar: 50 μm. Data are represented as the mean ± SD.

Three microarray expression profiles (GSE113439, GSE15197, and GSE53408) were downloaded from the GEO dataset, and the differentially expressed genes (DEGs) involved in PAH were screened according to the criteria of *P* value < 0.05 and |log_2_ (FC)| ≥1. As shown in Fig. 1a, 117 overlapping DEGs were found in these three datasets, including USP15. The GO and KEGG analyses were performed to determine the biological features of DEGs (Fig. 1b, c). We further collected lung tissues from PH patients and non-PH control individuals. As indicated in Fig. 1d, e, the protein levels of USP15, YAP1, and TAZ were augmented in the PH samples. Similar trends were also observed in the mRNA levels of USP15. However, no significant difference was found in the mRNA levels of YAP1 and TAZ between the control and PH groups. Moreover, images of immunofluorescence staining suggested that USP15 could colocalize with α-SMA in the pulmonary arteries of the PH patients (Fig. 1f), which indicates the potential regulatory role of USP15 in PASMCs.

### USP15 was upregulated in lung tissues of mice with PH

**Fig. 2:**
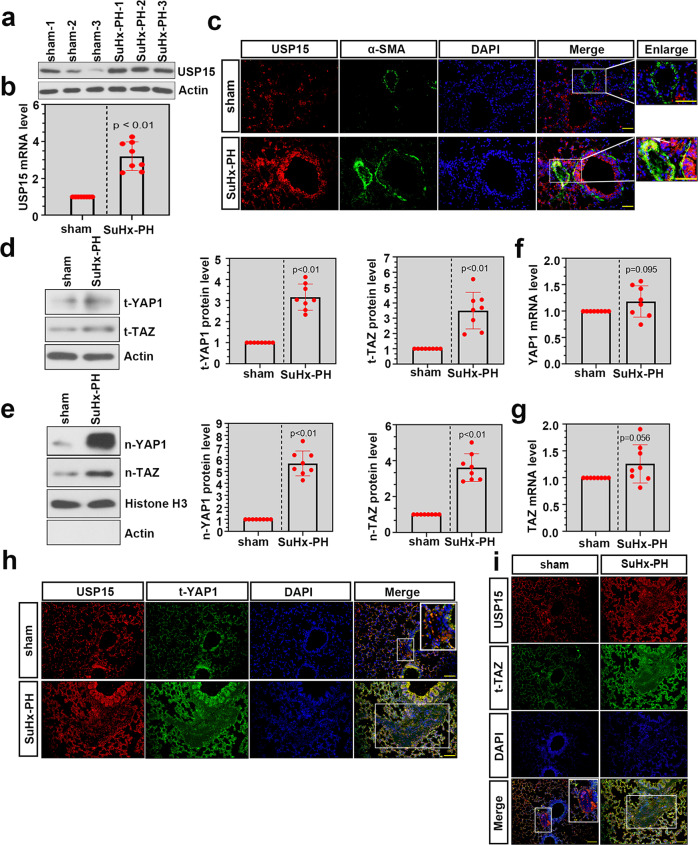
USP15 expression and YAP1/TAZ signaling were significantly increased in lung tissues of SuHx-PH mice. The SU5416/hypoxia (SuHx)-PH mouse model was used to mimic PH in vivo. **a**, **b** Real-time PCR and western blot analysis were performed to detect the pulmonary expression level of USP15 after modeling. **c** Representative immunofluorescence images of USP15 (red) and α-SMA (green) in lung tissues from the SuHx-PH mice and the sham mice. Scale bar: 50 μm. The white frame indicates the location of the pulmonary artery. **d**, **e** The protein levels of t-YAP1, t-TAZ, n-YAP1, and n-TAZ in the SuHx-PH and sham groups were determined by western blotting. **f**, **g** The mRNA levels of YAP1 and TAZ in lung tissue were detected by real-time PCR. **h** Coimmunostaining images of USP15 (red) and t-YAP1 (green) in lung sections from the sham and SuHx-PH groups. Scale bar: 100 μm. **i** Representative immunofluorescence results of USP15 (red) and t-TAZ (green) in lung sections from the sham and SuHx-PH groups. Scale bar: 100 μm. The white frame indicates the location of the pulmonary artery. Data are represented as the mean ± SD, *n* = 8 in each group.

USP15 expression in the lungs of mice with SuHx-induced PH was examined by western blotting and real-time PCR. As shown in Fig. 2a, b, USP15 expression was significantly augmented in lung tissues of the mice with experimental PH. Immunofluorescence staining was further conducted using USP15/α-SMA antibodies. USP15 colocalized with increased α-SMA in the SuHx-PH group (Fig. 2c), which suggests the expression of USP15 in PASMCs of the SuHx-PH mice. In addition, the SuHx-PH mice showed a significant increase in the pulmonary expression of total YAP1 (t-YAP1), total TAZ (t-TAZ), nuclear YAP1 (n-YAP1), and nuclear TAZ (n-TAZ) (Fig. 2d, e). However, no obvious difference was found in their mRNA levels after SuHx-PH modeling (Fig. 2f, g). The general distribution of pulmonary arteries on lung sections was determined by H&E staining, as shown in Supplementary Fig. 1. Immunofluorescence analysis was subsequently conducted on a serial section to identify the colocalization of USP15 with YAP1/TAZ in pulmonary arteries. As shown in Fig. 2h, i, USP15 positive staining (red) shared increased overlap with YAP1/TAZ staining after SuHx induction, which suggests that USP15 was colocalized with YAP1/TAZ in pulmonary arteries after SuHx-PH modeling.

### Knockdown of USP15 alleviated SuHx-induced PH exacerbation in mice

**Fig. 3:**
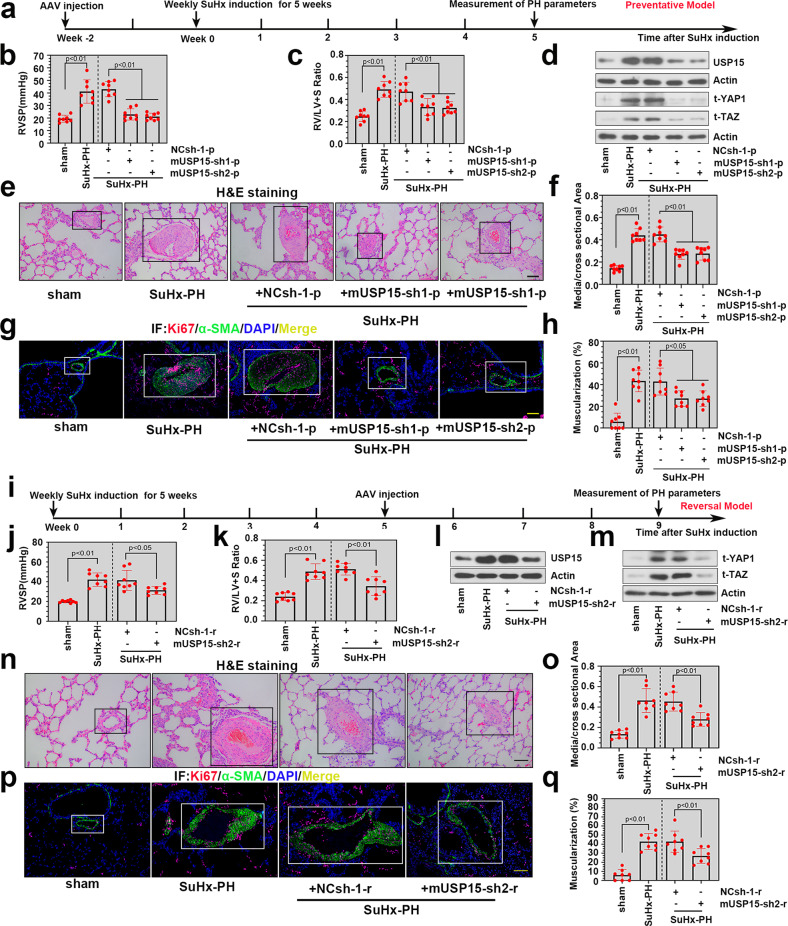
Knockdown of USP15 alleviated SuHx-induced PH exacerbation in mice. **a** In the preventative model, AAVs delivering shRNAs against mUSP15 were injected into mice 2 weeks prior to SuHx induction. **b** Right ventricular systolic pressure (RVSP) of mice was measured after modeling. **c** The ratio of RV/LV + S was calculated to evaluate right ventricular hypertrophy. **d** Expression levels of USP15, t-YAP1, and t-TAZ were further detected by immunoblot analysis after modeling. **e** Histological changes in pulmonary tissues were determined by H&E staining. The pulmonary artery in lung sections is indicated by a black square frame. (Scale bar: 100 μm). **f** Calculation of the ratio of vascular medial thickness to cross-sectional area (medial/cross-sectional area). **g** Representative immunofluorescence images of Ki-67 (red) and α-SMA (green) in lung tissues from each group. Scale bar: 100 μm. The white frame indicates the location of the pulmonary artery. **h** Quantitative analyses of muscularized pulmonary arterioles. **i** In the reversal model, AAVs were administered after SuHx induction. Four weeks after AAV intervention, RVSP (**j**) and the ratio of RV/LV + S (**k**) were measured. **l**, **m** The expression levels of USP15, t-YAP1, and t-TAZ were further detected by western blotting. **n** Representative H&E-stained lung sections from mice exposed to SuHx with/without AAV intervention. The pulmonary artery in lung sections is indicated by a black square frame. Scale bar: 100 μm. **o** Quantification of the medial/cross-sectional area. **p** Immunofluorescence images of Ki-67 (red) and α-SMA (green) in sections from mice exposed to SuHx with/without AAV intervention. Scale bar: 100 μm. The white frame indicates the location of the pulmonary artery. **q** Quantification of muscularized pulmonary arterioles in mice exposed to SuHx with AAV intervention. Data are represented as the mean ± SD, *n* = 8 in each group.

To explore the preventative effect of USP15 knockdown on SuHx-induced PH, we injected AAVs carrying mUSP15/NC shRNAs into the experimental mice 14 days before SuHx induction (Fig. 3a). Notably, preventative knockdown of USP15 markedly reduced RVSP and the RV/LV + S ratio in the SuHx-induced PH mice (Fig. 3b, c). USP15 silencing in vivo was validated in Fig. 3d, and it significantly reduced the expression levels of YAP1 and TAZ in the SuHx-PH group (Fig. 3d). Pulmonary vascular wall thickening was measured by H&E staining. The SuHx-PH group showed an obviously thickened pulmonary vascular wall compared to the sham group, while preventative knockdown of USP15 remarkably alleviated this change (Fig. 3e). Similar trends were determined in the ratio of media/cross-sectional area (Fig. 3f). To further investigate the effect of USP15 knockdown on PASMC proliferation in vivo, we confirmed the infectivity of AAVs on PASMCs in vivo, as shown in Supplementary Fig. 2. Double immunofluorescence staining was subsequently carried out using Ki-67/α-SMA antibodies. As revealed in Fig. 3g, the proportion of Ki-67^+^/α-SMA^+^ cells was significantly increased in the SuHx-PH group and decreased after the preventative knockdown of USP15. The degree of vascular muscularization was evaluated based on α-SMA staining in lung sections, and the percentage of muscularized pulmonary arterioles was markedly increased after SuHx-PH modeling and decreased after USP15 silencing (Fig. 3h). In the reversal model of PH, AAVs were injected into the SuHx-treated mice after 5 weeks of PH modeling (Fig. 3i). Interestingly, knockdown of USP15 evidently reduced RVSP and the RV/LV + S ratio in the reversal model of SuHx-induced PH (Fig. 3j, k), and it decreased the abundance of YAP1/TAZ in lung tissues of the SuHx-PH mice (Fig. 3l, m). Additionally, the reversal effect of USP15 knockdown on pulmonary vascular wall thickening and PASMC proliferation in the SuHx-PH mice was validated in Fig. 3n–q, as evidenced by a decreased ratio of media/cross-sectional area, less vascular muscularization, and reduced Ki-67 expression.

### Knockdown of USP15 alleviated MCT-induced PH exacerbation in rats

**Fig. 4:**
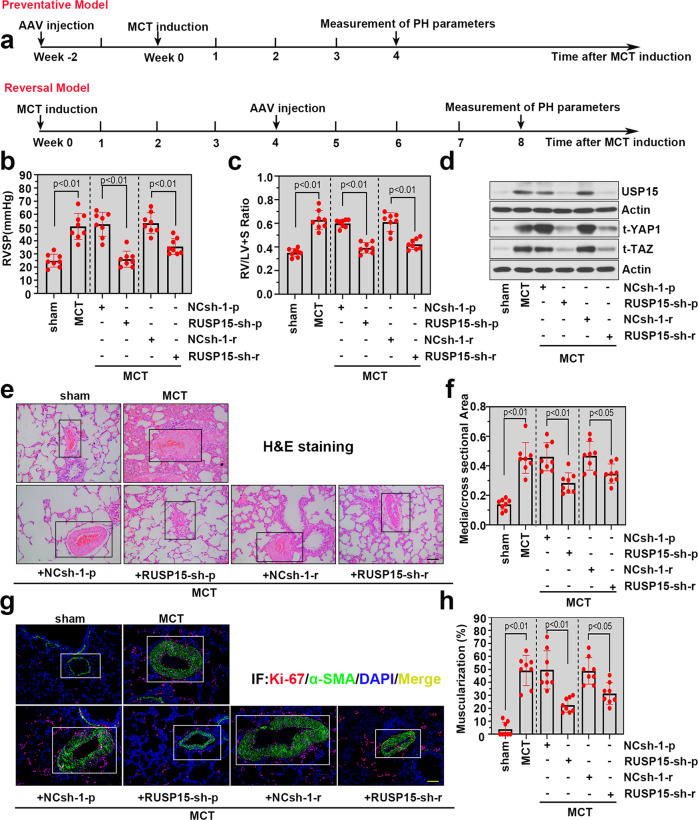
Knockdown of USP15 alleviated MCT-induced PH exacerbation in rats. **a** An additional PH model was established in SD rats by MCT challenge. In the preventative model, AAVs carrying shRNA against RUSP15 were injected into rats 14 days before MCT induction. In the reversal model, rats received AAV injection after 4 weeks of MCT challenge. RVSP (**b**) and the ratio of RV/LV + S (**c**) were measured in both preventative and reversal models. **d** Western blot analysis was conducted to determine the expression levels of USP15, t-YAP1 and t-TAZ in lung tissues from each group. **e** Representative H&E-stained lung sections in both the preventative and reversal models. The pulmonary artery in lung sections is indicated by a black square frame. Scale bar: 100 μm. **f** Quantification of the medial/cross-sectional area. **g** Representative immunofluorescence images of Ki-67 (red) and α-SMA (green) in lung sections from the MCT-challenged rats with/without AAV intervention. Scale bar: 100 μm. The white frame indicates the location of the pulmonary artery. **h** Quantification of muscularized pulmonary arterioles. Data are represented as the mean ± SD, *n* = 8 in each group.

We then investigated the preventative/reversal effect of USP15 knockdown in rats with MCT-induced PH. The experimental workflow is presented in Fig. 4a. Consistent with the SuHx-induced PH model, MCT challenge significantly elevated RVSP and the RV/LV + S ratio in SD rats, while preventative/reversal knockdown of USP15 markedly attenuated these pathological parameters (Fig. 4b, c). The downregulation of USP15, YAP1, and TAZ was validated after USP15 knockdown in a preventative/reversal model of PH (Fig. 4d). In addition, USP15 knockdown significantly alleviated pulmonary vascular wall thickening in the MCT-treated rats (Fig. 4e, f). Ki-67 expression in PASMCs in vivo was further determined by double immunofluorescence staining. USP15 knockdown in the preventative/reversal model markedly decreased the proportion of Ki-67^+^/α-SMA^+^ cells in the MCT group, which suggests the inhibitory effect of USP15 knockdown on PASMC proliferation (Fig. 4g). Moreover, similar trends were observed in vascular muscularization (Fig. 4h). Significantly less vascular muscularization was found after USP15 knockdown in both the preventative/reversal models of MCT-induced PH.

### USP15 expression was highly induced in hypoxia-treated hPASMCs

**Fig. 5:**
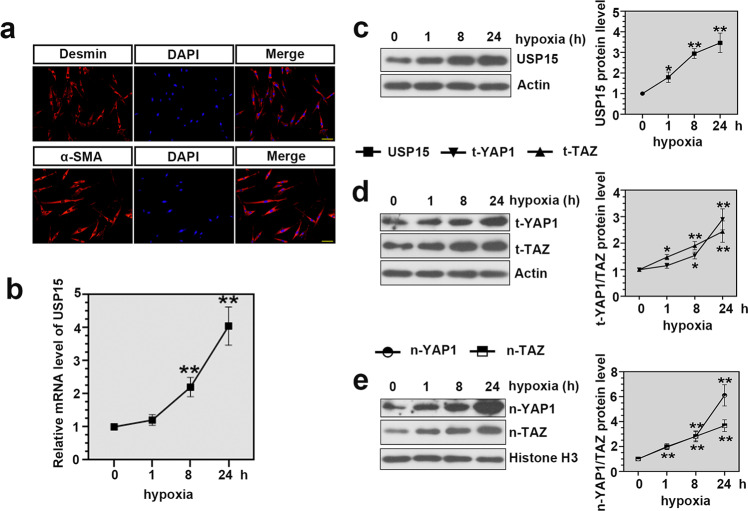
Expression of USP15 and YAP1/TAZ signaling was significantly increased in hypoxia-treated hPASMCs. **a** Identification of hPASMCs by immunofluorescence staining. Scale bar: 100 μm. **b**, **c** The mRNA and protein levels of USP15 in the hypoxia-treated hPASMCs. **d** The protein levels of t-YAP1 and t-TAZ in the hypoxia-treated hPASMCs. **e** The protein levels of n-YAP1 and n-TAZ in the hypoxia-treated hPASMCs. Data are represented as the mean ± SD, *n* = 4 in each group. Compared with values at 0 h, *P < 0.05, **P < 0.01.

Immunofluorescence staining was performed to determine the positive staining of α-SMA and desmin in hPASMCs (Fig. 5a). Cells were then exposed to hypoxia for 0, 1, 8, and 24 h. As expected, the expression level of USP15 progressively increased following hypoxia treatment (Fig. 5b, c). In addition, significantly augmented expression levels of t-YAP1, t-TAZ, n-YAP1, and n-TAZ were identified in the hypoxia-induced hPASMCs (Fig. 5d, e).

### USP15 deficiency inhibited cell proliferation, migration, and YAP1/TAZ signaling in hypoxia-induced hPASMCs

**Fig. 6:**
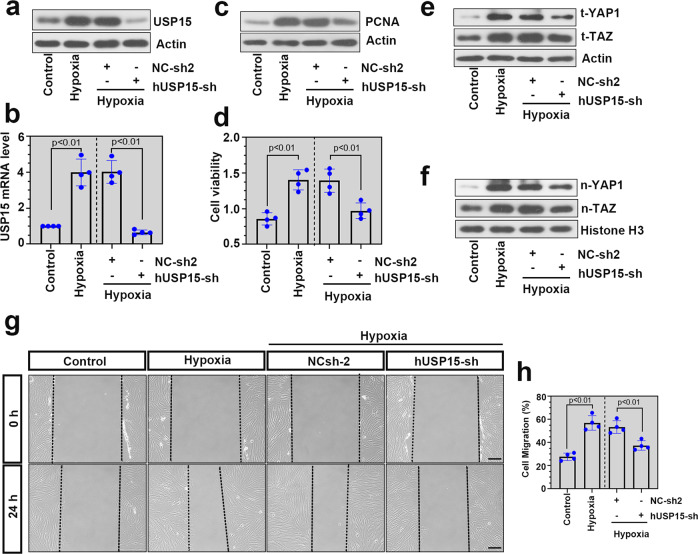
USP15 deficiency inhibited cell proliferation, migration, and YAP1/TAZ signaling in hypoxia-induced hPASMCs. Adenovirus delivering shRNA against hUSP15 was used to downregulate USP15 expression in hPASMCs. After 48 h of infection, cells were cultured under hypoxia for another 24 h. The protein level (**a**) and mRNA level (**b**) of USP15 in the treated cells were measured by western blotting and real-time PCR, respectively. **c** The abundance of PCNA protein was detected by western blot analysis. **d** A CCK-8 assay was performed to determine cell viability after treatments. **e**, **f** The protein levels of t-YAP1, t-TAZ, n-YAP1, and n-TAZ in the treated cells were measured by western blotting. **g**, **h** Cell migration in each group was determined by wound healing assay. Scale bar: 200 μm. Data are represented as the mean ± SD, *n* = 4 in each group.

We next investigated the effect of USP15 on cell proliferation and migration in hypoxia-induced hPASMCs. Loss of functional USP15 was achieved in hPASMCs by Ad-hUSP15-sh infection. As shown in Fig. 6a, b, USP15 was predominantly upregulated in the hypoxia-treated cells and downregulated after infection. CCK-8 assays and immunoblotting were performed to determine the effect of USP15 on the proliferation of the hypoxia-induced hPASMCs. As revealed in Fig. 6c, d, USP15 knockdown significantly inhibited excessive proliferation in the hypoxia-treated hPASMCs, which was manifested by decreased PCNA expression and reduced cell viability. In addition, the protein levels of t-YAP1, t-TAZ, n-YAP1, and n-TAZ were decreased in the hypoxia-treated cells after USP15 silencing (Fig. 6e, f). hPASMCs were next subjected to a wound healing assay after infection, and USP15 deficiency exhibited an inhibitory effect on the migration of the hypoxia-stimulated cells (Fig. 6g, h).

### USP15 overexpression promoted hPASMC proliferation and migration in a YAP1/TAZ-dependent manner

**Fig. 7:**
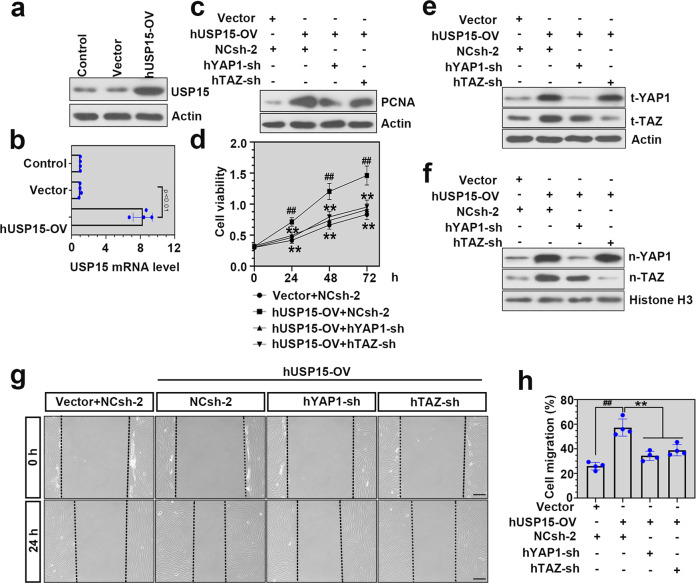
Overexpression of USP15 promoted hPASMC proliferation and migration in a YAP1/TAZ-dependent manner. Adenovirus delivering overexpressed hUSP15 was used to upregulate USP15 expression in hPASMCs under normoxia. Western blot analysis (**a**) and real-time PCR (**b**) were conducted to determine the infectivity of Ad-hUSP15-OV after 72 h of infection. Afterward, hPASMCs were coinfected with adenovirus delivering overexpressed hUSP15 and hYAP1-sh or hTAZ-sh. **c** The expression level of PCNA in hPASMCs was detected by immunoblot analysis after coinfection. **d** A CCK-8 assay was performed to detect the viability of hPASMCs after coinfection. **e**, **f** The expression levels of t-YAP1, t-TAZ, n-YAP1, and n-TAZ in hPASMCs were detected by western blotting after coinfection. **g**, **h** Cell migration in hPASMCs was determined by wound healing assays after coinfection. Scale bar: 200 μm. Data are represented as the mean ± SD, *n* = 4 in each group. Compared to the Vector+NCsh-2 group, ^#^*P* < 0.05, ^##^*P* < 0.01; compared to the hUSP15-OV + NCsh-2 group, *P < 0.05, **P < 0.01.

To further explore whether USP15 mediated hPASMC proliferation and migration in a YAP1/TAZ-dependent manner, we achieved the depletion of YAP1 or TAZ in USP15-overexpressing hPASMCs by corresponding adenovirus infection. As shown in Fig. 7a, b, the overexpression of USP15 was determined by western blotting and real-time PCR. PCNA expression and cell viability in the USP15-overexpressing hPASMCs were remarkably inhibited after the knockdown of YAP1 or TAZ (Fig. 7c, d). Moreover, the USP15-overexpressing hPASMCs displayed increased expression levels of t-YAP1, t-TAZ, n-YAP1, and n-TAZ compared to the controls, while they were reduced after YAP1/TAZ knockdown (Fig. 7e, f). A wound-healing assay was conducted to determine cell migration in hPASMCs after infection. We confirmed that hPASMC migration was significantly increased after USP15 overexpression and reduced after the knockdown of YAP1 or TAZ (Fig. 7g, h).

### USP15 promoted YAP1 stability by inhibiting K48-linked ubiquitination of YAP1

**Fig. 8:**
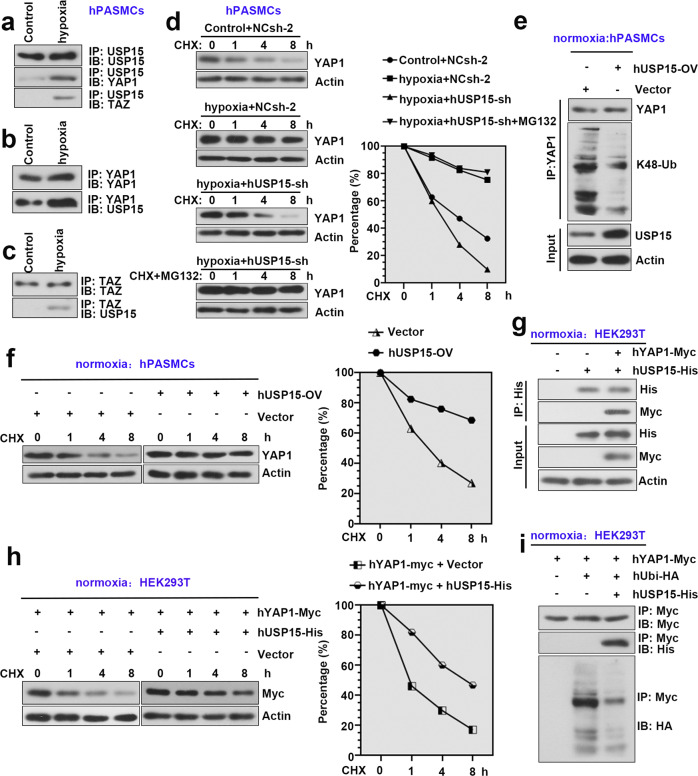
USP15 promoted YAP1 stability by inhibiting K48-linked ubiquitination of YAP1. **a** Anti-USP15 immunoprecipitation was used to determine the interaction between USP15 and YAP1 or TAZ in the control and hypoxic hPASMCs. **b** Anti-YAP1 immunoprecipitation to determine the interaction between YAP1 and USP15 in the control or hypoxic hPASMCs. **c** Anti-TAZ immunoprecipitation to determine the interaction between TAZ and USP15 in the control or hypoxic hPASMCs. **d** hPASMCs were infected with Ad-hUSP15-sh or Ad-NCsh-2 for 48 h. After 3 h of starvation, cells were exposed to hypoxia for 24 h and treated with CHX (25 μg/ml) or MG132 (5 μM) for 0, 1, 4, or 8 h (at the last 8 h of hypoxic time). Cell samples were harvested for immunoblot analysis to detect YAP1 expression. The percentage of YAP1/actin is shown in the right panel. **e** hPASMCs infected with Ad-hUSP15-OV or Ad-Vector were treated with MG132 (5 μM) under normoxia for 8 h, and cells were harvested for immunoprecipitation using anti-YAP1 antibody. **f** Under normoxia, infected hPASMCs were subjected to a CHX pulse-chase assay. Cell samples were harvested for immunoblot analysis to detect YAP1 expression. The percentage of YAP1/Actin is shown in the right panel. **g** His-tagged USP15 and Myc-tagged YAP1 were cotransfected into HEK293T cells. At 48 h post-transfection, cell samples were harvested for immunoprecipitation using an anti-His antibody. **h** HEK293T cells were cotransfected with Myc-tagged YAP1 and His-tagged USP15/vector. At 48 h post-transfection, cells were treated with CHX (25 μg/ml) for 0, 1, 4, and 8 h. YAP1 expression in cell samples was determined by western blot analysis using an anti-Myc antibody. The percentage of YAP1/actin is shown in the right panel. **i** His-tagged USP15, HA-tagged Ubi and Myc-tagged YAP1 were cotransfected into HEK293T cells. At 48 h post-transfection, cells were treated with MG132 (5 μM) for 5 h. Cell samples were then harvested for immunoprecipitation using anti-Myc antibody. Data are represented as the mean ± SD, *n* = 4 in each group.

To determine the functional association between USP15 and YAP1/TAZ, we performed coimmunoprecipitation analyses. As shown in Fig. 8a–c, endogenous USP15 could interact with YAP1 in the control and hypoxic hPASMCs, while the interaction between USP15 and TAZ was observed upon hypoxia treatment. A CHX chase assay was performed to determine the effect of USP15 on YAP1 stability in the hypoxic hPASMCs. As shown in Fig. 8d, YAP1 protein expression was progressively decreased after USP15 knockdown in the hypoxia-induced hPASMCs, while its expression was significantly elevated after treatment with MG132, an inhibitor of the ubiquitin-proteasome pathway. We thus investigated the effect of USP15 on the ubiquitination level of YAP1. As shown in Fig. 8e, USP15 distinctly inhibited K48-linked ubiquitination of YAP1 in normoxic hPASMCs. The CHX chase assay further determined that USP15 promoted the protein stability of YAP1 in normoxic hPASMCs (Fig. 8f). Additionally, His-tagged USP15 and Myc-tagged YAP1 plasmids were cotransfected into HEK293T cells. Cell samples were next subjected to immunoprecipitation and CHX chase assays. As shown in Fig. 8g, His-tagged USP15 interacted with Myc-tagged YAP1. In addition, His-tagged USP15 dramatically promoted the protein stability of Myc-tagged YAP1 by inhibiting the ubiquitination of YAP1 (Fig. 8h, i). Taken together, our findings indicate that USP15 might promote PH progression in a YAP1/TAZ-dependent manner (Supplementary Fig. 3).

## Discussion

PH is a fatal cardiopulmonary disease with multiple risk factors. Recently, major efforts have been devoted to elucidating the pathogenesis underlying PH. However, the molecular mechanisms of PH remain poorly explored. In the present study, we first identified the upregulation of USP15 in the lungs of PH patients and experimental PH animal models. The preventative/reversal knockdown of USP15 in vivo evidently inhibited pulmonary vascular wall thickening, right ventricular hypertrophy, and YAP1/TAZ signaling in animals with experimental PH. We further determined that USP15 promoted PASMC proliferation and migration in a YAP1/TAZ-dependent manner, which suggests that USP15 might be a vital regulator of PH.

USP15 is an essential DUB that functions by removing ubiquitin chains from its target proteins. This enzyme has been shown to play an essential role in several biological processes, such as disease-related inflammation and tumorigenesis^11,28,29^. Interestingly, in this study, we found that USP15 expression was aberrantly regulated in the lungs of PAH patients after analyzing microarray profiling data from the GEO dataset. The results of GO analysis showed significant enrichment in several GO terms, such as vesicle transport along an actin filament, DNA repair, and mitotic spindle pole, which provides us with valuable information for further study. In addition, KEGG pathway analysis suggested obvious enrichment in hypertrophic cardiomyopathy and dilated cardiomyopathy, two disorders associated with PH^30,31^. Our clinical results validated the upregulation of USP15 in lung tissues from PH patients, further indicating the potential involvement of USP15 in PH. In agreement with our clinical data, USP15 was significantly upregulated in lung tissues from mice with SuHx-induced PH and rats with MCT-induced PH, two classical rodent experimental models of PH^32,33^. Surprisingly, we found that USP15 knockdown could alleviate PH symptoms in SuHx-induced mice and MCT-induced rats, as evidenced by the decreased pulmonary artery wall thickness and reduced ratio of RV/LV + S. Previous studies have validated that SuHx induction and MCT challenge in vivo could result in obvious pulmonary artery wall thickening, a key feature of remodeled vasculature^34^. In addition, PH is a severe disease that can progressively develop into right ventricular failure^35^. Accordingly, in this study, obvious right ventricular hypertrophy manifested as a vastly elevated RV/LV + S ratio was validated after SuHx and MCT induction, and it was significantly alleviated after USP15 knockdown.

USP15 was further found to be colocalized with increased α-SMA in response to PH modeling. The accumulation of α-SMA is a general discovery in remodeled arteries, and it is a key marker of smooth muscle cell (SMC) hypertrophy^36^. PASMCs are important contributors to pulmonary arterial remodeling during PH development^37^. PASMCs with PH always display several abnormal properties, such as uncontrolled cell growth and excessive migration^38^. In this study, hypoxia-exposed PASMCs showed obviously promoted cell proliferation and migration when compared to the controls. In contrast, USP15 knockdown abrogated these abnormalities in hypoxic cells, which suggests that USP15 deficiency might protect against pulmonary arterial remodeling in PH by inhibiting PASMC proliferation and migration.

YAP1 and TAZ are two central transcriptional regulators of the Hippo pathway, which has been shown to be critically involved in organ size control and tissue homeostasis^39–41^. In addition, YAP1/TAZ signaling is activated in cardiac tissues after injury, and it has been a promising target for preventing abnormal remodeling in cardiac diseases^42^. Notably, YAP1/TAZ activity is extremely elevated in experimental PH and induces remodeling phenotypes in PASMCs^20^. Notably, a similar observation was made in this study. The expression levels of t-YAP1, t-TAZ, n-YAP1 and n-TAZ were markedly increased in the lung tissue of mice with SuHx-induced PH, rats with MCT-induced PH, and hypoxia-induced PASMCs, while USP15 knockdown inhibited YAP1/TAZ signaling in these experimental PH models by restraining their expression and nuclear translocation. Thus, we propose that USP15 might be a novel regulator of YAP1/TAZ signaling during PH progression.

Our rescue assay determined that USP15 promoted the proliferation and migration of hypoxia-exposed PASMCs in a YAP1/TAZ-dependent manner. We thus investigated the functional association between USP15 and YAP1/TAZ. USP15 interacted with YAP1 in PASMCs exposed to normoxia or hypoxia. However, an interaction between USP15 and TAZ was observed under hypoxia. Although YAP1 and TAZ are equally important downstream effectors of the Hippo pathway, TAZ is much more unstable than YAP1, with a shorter half-life of 2 h^43,44^. Additionally, YAP1 has been documented to regulate PASMC proliferation during PH progression^22,45^. Therefore, YAP1 was selected as the major subject for further study.

As previously described, DUBs are capable of removing ubiquitin from their substrates and promoting their abundance level. USP15, a representative DUB, has been documented to exhibit regulatory effects on many disorders through its deubiquitination ability. Accordingly, USP15 facilitates cerebellar homeostasis by deubiquitinating TUT1^46^. USP15 regulates cutaneous wound repair by inhibiting the ubiquitination of EIF4A1^47^. We, therefore, explored whether USP15 contributes to PH development by deubiquitinating YAP1 and found that USP15 promoted the stability of YAP1 by inhibiting K48-linked ubiquitination of YAP1. Ubiquitination is a crucial type of post-translational modification that facilitates the linkage of ubiquitin to target proteins. Researchers have identified 8 different types of ubiquitination, such as K48/K63/K11-linked ubiquitination^48^. Notably, the physiological role of K48-linked ubiquitin chains has been well established, and it contributes to the proteasomal degradation of target proteins^48^.

In summary, this study identified the upregulation of USP15 in the lungs of PH patients and experimental PH models. Conversely, loss of functional USP15 significantly alleviated PH exacerbation in SuHx-treated mice and MCT-challenged rats. USP15 facilitated the proliferation and migration of hypoxic hPASMCs in a YAP1/TAZ-dependent manner, and it promoted the stability of YAP1 by inhibiting YAP1 ubiquitination. Taken together, our findings highlight the regulatory role of USP15 in PH progression and provide references for the investigation of PH pathogenesis.

## Supplementary information

Supplementary information
