# Supplementary material for: USP15 promotes pulmonary vascular remodeling in pulmonary hypertension in a YAP1/TAZ-dependent manner
Source: Exp Mol Med. 2023 Jan 12;55(1):183–95. doi: 10.1038/s12276-022-00920-y (PMC9898287; doi:10.1038/s12276-022-00920-y)

Supplementary information

Supplementary Fig. 1

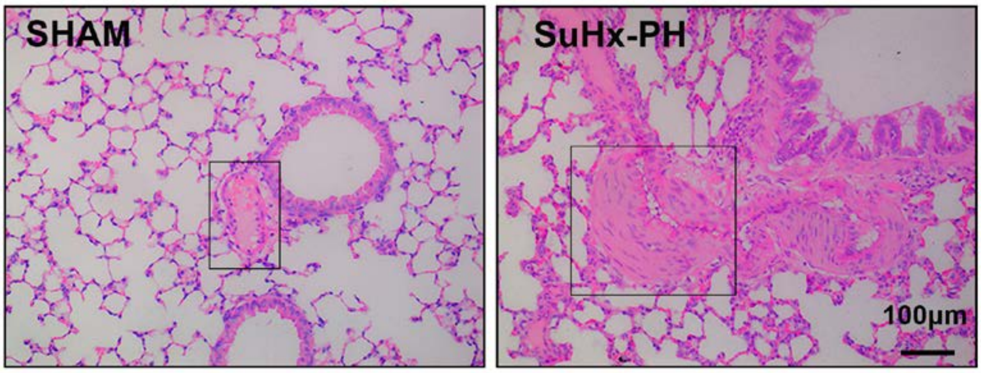

Supplementary Fig. 1 Histological analysis on lung sections from sham and SuHx-induced PH mice.

The representative images of H&E-stained lung section in sham and SuHx-PH group. Black square frame in indicated the location of pulmonary artery. Scale bar: 100 μm.

Supplementary Fig. 2

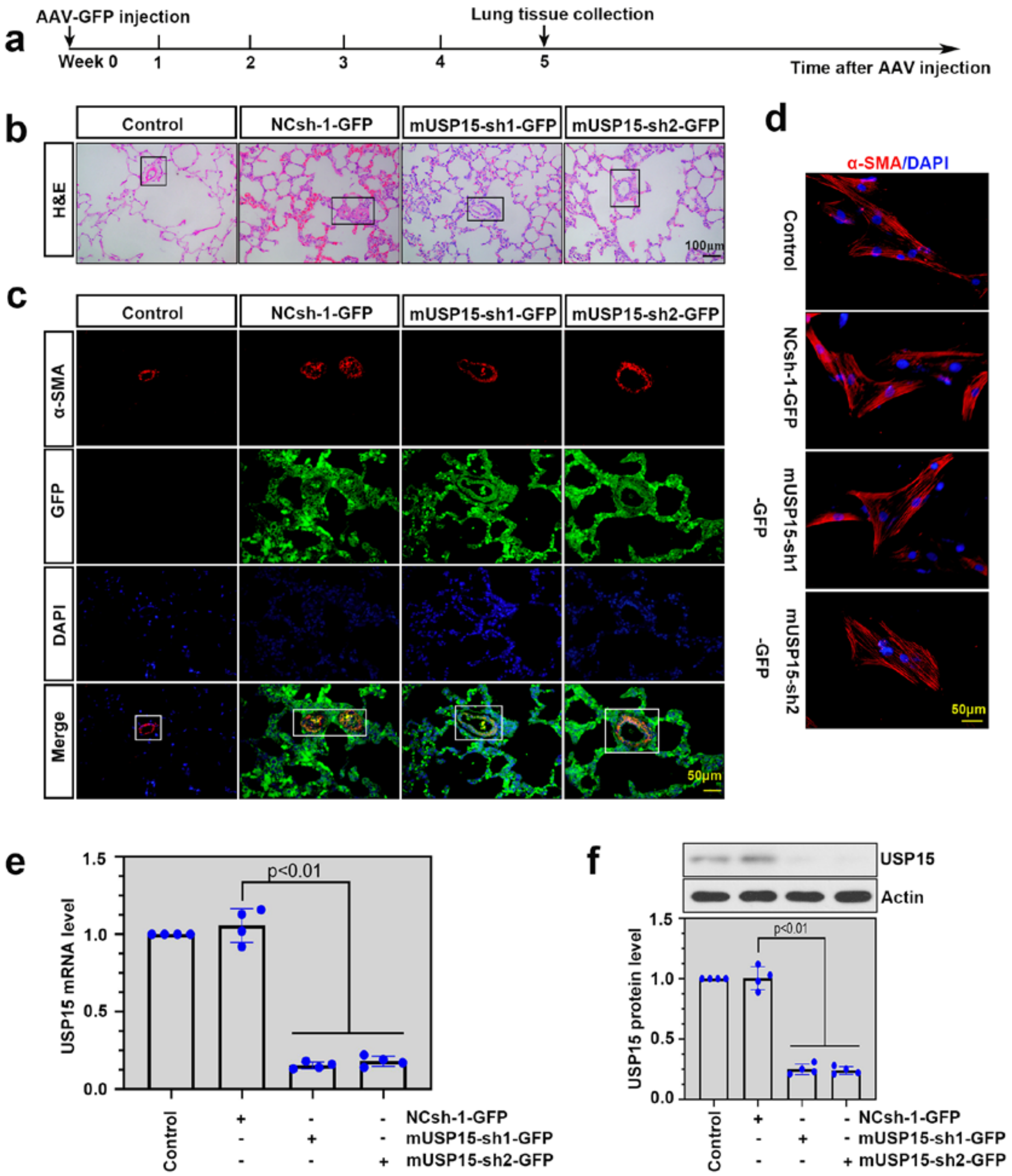

Supplementary Fig. 2 Identification of AAV infectivity in PASMCs in vivo

(a) AAV expression vector with GFP tag was used to generate AAVs expressing NCsh1, mUSP15-sh1 and mUSP15-sh2. There are no difference between this GFP-tagged vector and the previous one except for the GFP tag. Corresponding AAVs-GFP (1×10^{12} vector genomes/ml) was intravenously injected into C57BL/6 mice for 5 weeks. (b) Lung sections were subjected to H&E staining. Pulmonary

artery in lung sections was indicated by black square frame. Scale bar: 100 μm. (c) Double immunofluorescence staining was carried out to determine the co-localization of α-SMA (red) and AAV-GFP (green). Scale bar: 50 μm. Of note, H&E staining and immunofluorescence staining were performed on serial sections. (d) Primary PASMCs were isolated from each group and identified by immunofluorescence staining using α-SMA antibody. Scale bar: 50 μm. The mRNA level (e) and protein level (f) of USP15 in isolated PASMCs was detected by real-time PCR and western-blot.

Supplementary Fig. 3

Graphic abstract of USP15 regulation on PH development

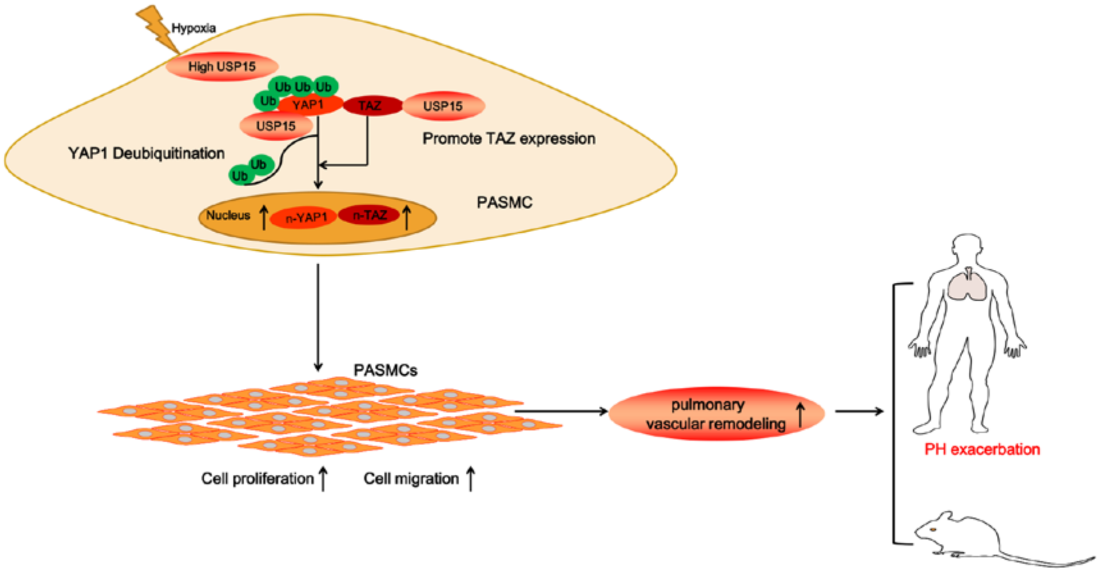

Supplement: Supplementary file 1 — Supplementary information [file 12276_2022_920_MOESM1_ESM.pdf]
